# Potentiation Physiologically Proven

**Published:** 1893-12

**Authors:** Gustav Jaeger

**Affiliations:** Stuttgart, Germany


### POTENTIATION PHYSIOLOGICALLY PROVEN.

PROF. DR. GUSTAV JAEGER, STUTTGART, GERMANY.

[Translated by B. Fincke, M. D. Concluded from page 544.]

Hahnemann says in his *Organon* (5th ed., p. 295, note): "The higher the dilution combined with potentiation (by two shaking strokes) is carried the more rapid and penetrating the preparation seems to alter the life-force medicinally, and to change the state, with only lightly lessened strength, even when the process is carried very far—instead of (and mostly sufficient) to X as usual, to XX, L, C, and higher; only that then the action always seems to last a shorter time."

Aside from the last short sentence my neuro-analytical result, therefore, is nothing else than what *Hahnemann* has taught and I am not at all an *innovator*, but only a *renovator*, in so far as in the Neural Analysis I have found a means of "proving by numbers" what Hahnemann taught, and numbers have a stronger argumentative power than words.

Then, I should like to point to the American colleagues, with whom, according to what I hear, the high potency, especially the 200th potency, enjoy a much greater popularity than with us. When we see how the American art and industry accomplishes the most perfect on all the fields where it appears in competency

—I mention only the American stoves, the American dentistry—this difference in our fields seems to me to depend upon the following :

In America the only driving power is the success. This drives the American colleagues from potency to potency. In Germany the Hahnemannian doctrine of potentiation succumbed to the overpowering pressure of the *scholastics* with its infallible *dogmas* protected by the governments. Our homœopathic physicians were forced to submit to this paralyzing influence of the schoolmen in their youth and to pay to it the *sacrificium intellectus.* The fruits of these scholastics persecute them in *praxi* to the grave; there is no use of the *success at the bedside of the sick,* with it in our scholastically corrupted home one gains not "respect," but only "envy." In America the "success determines the respect," with us it is conditioned by the "examen" besides "title" and "rank;" of course our bureaucracy does not allow any shaking up of this state of things because it is in the same boat.

And now a last word :

Already in the first section of my publication I have said: what I need in a possible continuation of my work, respectively its publication, are "co-operators," and what I do not need are "criticasters," and the same is also valid for the reader. In relation to both I should like to make a few remarks:

Co-operators in this matter can be sure that I always shall be at their service with the experience which I have gained in a practice of long years and that in this direction I shall shun neither time nor labor.

For "criticasters," however, I am not at home, especially not for that numerous host who at every occasion throw in their objection "imagination!" In my life of now sixty years I have had enough opportunity to recognize these people punished with this imagination that a demonstration with them is as useless as a conversation with a post. They differ from such a one only by their being able to talk and write, for they use these faculties merely to maintain their position as post which must not be removed. With a post only two things can be done, either it must be avoided or it must be cut down. Since the latter is not respectable I prefer the former.

Singular: these people deem themselves enormously wise and have no inkling that in comparison with them a Don Quixote is a sublime figure. This man was at least a *knight* of *imagination*, but they are no knights, they can neither ride on horseback nor fight, they are only *posts of imagination*. The comical part of it is especially that these imaginative fellows do not understand anything about imagination, have nothing of it, and cannot do anything with it. Whilst imagination is one of the strongest powers in human life, they unite with it the conception of a "nothing," of a thing about which we need not care. Their idea of "imagination" prevents them on one hand from learning anything, on the other hand they do not even know what to do with it. Imagination is only a power if one believes in what one imagines, and there is the rub; something which one takes for *nothing* one cannot believe. Yea, the matter is still more foolish; these creatures afflicted with imagination cannot even imagine something, because the image immediately dissolves into nothing if it is taken for imagination. In other words, only in the hand of a man of *belief* imagination is a weapon, in the hand of a skeptic it is air, nothing; and such a one is a nihilist in the most audacious meaning of the word: he has nothing and he can do nothing.

If one thinks, the skeptic has of his skepticism the advantage that he lives to see no disillusion, this is twice false. There are only two cases: either a matter is imagination or not. In the last case the skeptic lives to see the great disillusion that the go-aheader (Draufgänger) attains to advantage much sooner than he, and this is for him not only a disillusion but a disadvantage. In the first case he experiences of course no disillusion, but whilst on the whole he experiences nothing because he does nothing, the one who follows his imagination experiences in the worst case *disadvantage*, and *damage makes wise*, for even if one follows an imagination one makes a whole quantity of valuable experiences, whilst the skeptic, because he does nothing, also experiences nothing and becomes no wiser than he was. The skeptics are the dry branches on the tree of life.

